# Supplementary material for: Benzoxazinoids Biosynthetic Gene Cluster Identification and Expression Analysis in Maize under Biotic and Abiotic Stresses
Source: Int J Mol Sci. 2024 Jul 7;25(13):7460. doi: 10.3390/ijms25137460 (PMC11242666; doi:10.3390/ijms25137460)
Supplement: Supplementary file 1 [file ijms-25-07460-s001.zip › Supplementary Materials/Supplementary Figure S1.pdf]

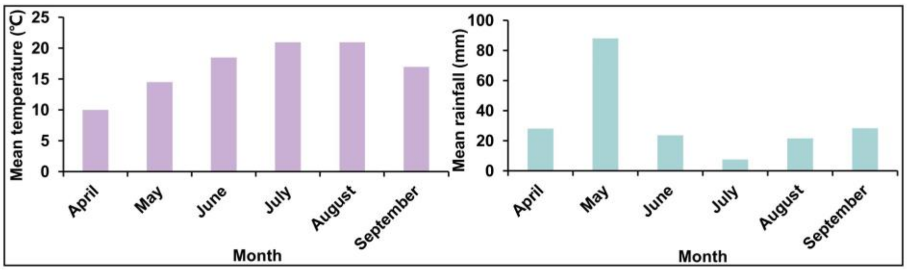

Figure S1. Weather data in all maize life stages at the Longxi experimental stations, Gansu, China (34°58′N, 104°23′E, 2,074 m altitude), 2023.
